# GPR119 agonist enhances gefitinib responsiveness through lactate-mediated inhibition of autophagy

**DOI:** 10.1186/s13046-018-0949-2

**Published:** 2018-11-29

**Authors:** Ji Hye Im, Keon Wook Kang, Sun Young Kim, Yoon Gyoon Kim, Yong Jin An, Sunghyouk Park, Byung Hwa Jeong, Song-Yi Choi, Jin-Sun Lee, Keon Wook Kang

**Affiliations:** 1 College of Pharmacy and Research Institute of Pharmaceutical Sciences, Seoul National University, Seoul, Republic of Korea; 2 Department of Nuclear Medicine, College of Medicine, Seoul National University, Seoul, Republic of Korea; 3 College of Pharmacy, Dankook University, Cheonan-si, Republic of Korea; 4 Natural Product Research Institute, College of Pharmacy, Seoul National University, Seoul, Republic of Korea; 5 Molecular Recognition Research Center, Korea Institute of Science and Technology, Seoul, Republic of Korea; 6 College of Medicine, Chungnam National University, Daejeon, Republic of Korea

**Keywords:** Autophagy, Breast cancer, Gefitinib, GPR119 agonist, Lactate

## Abstract

**Background:**

Ligand-dependent activation of the G-protein coupled receptor 119 (GPR119) lowers blood glucose via glucose-dependent insulin secretion and intestinal glucagon-like peptide-1 production. However, the function of GPR119 in cancer cells has not been studied.

**Methods:**

GPR119 expression was assessed by real-time qPCR and immunohistochemistry in human breast cancer cell lines and breast cancer tissues. Cell proliferation and cell cycle analyses were performed by Incucyte® live cell analysis system and flow cutometry, respectively. Autophagy activity was estimeated by western blottings and LC3-GFP transfection.

**Results:**

mRNA or protein expression of GPR119 was detected in 9 cancer cell lines and 19 tissue samples. Cotreatment with GPR119 agonist (MBX-2982 or GSK1292263) significantly potentiated gefitinib-induced cell growth inhibition in gefitinib-insensitive MCF-7 and MDA-MB-231 breast cancer cells. We observed that caspase-3/7 activity was enhanced with the downregulation of Bcl-2 in MCF-7 cells exposed to MBX-2982. Gefitinib-induced autophagy is related with cancer cell survival and chemoresistance. GPR119 agonists inhibit gefitinib-induced autophagosome formation in MCF-7 and MDA-MB-231 cells. MBX-2982 also caused a metabolic shift to enhanced glycolysis accompanied by reduced mitochondrial oxidative phosphorylation. MBX-2982 increased intracellular (~ 2.5 mM) and extracellular lactate (~ 20 mM) content. Gefitinib-mediated autophagy was suppressed by 20 mM lactate in MCF-7 cells.

**Conclusions:**

GPR119 agonists reduced mitochondrial OXPHOS and stimulated glycolysis in breast cancer cells, with consequent overproduction of lactate that inhibited autophagosome formation. Because autophagy is crucial for the survival of cancer cells exposed to TKIs, GPR119 agonists potentiated the anticancer effects of TKIs.

**Electronic supplementary material:**

The online version of this article (10.1186/s13046-018-0949-2) contains supplementary material, which is available to authorized users.

## Background

Anti-epidermal growth factor receptor (EGFR) therapy is an effective way to inhibit proliferation of many cancer types such as non–small cell lung cancer (NSCLC) and colorectal cancer [1]. However, EGFR-tyrosine kinase inhibitor (TKI) alone or in combination therapy with paclitaxel or docetaxel had been used for breast cancer patients in clinical trials, but both therapies failed [2]. Thus, it may be necessary to investigate the chemoresistance mechanism(s) to EGFR TKI for breast cancer patients.

Macroautophagy or autophagy is a lysosomal degradative process that recycles cellular components and maintains cell homeostasis [3]. In autophagy, double-membrane vesicles (autophagosomes) sequestrate selected substrates and fuse with lysosomes (autolysosomes). Autophagy dysfunction results in the accumulation of intracellular damaged proteins, causing neurodegeneration or cardiac hypertrophy [4]. The effect of autophagy activation on a cancer cell survival is controversial. Accelerated autophagy is believed to be an anticancer mechanism for a variety of agents. Nur77 agonists induce autophagic cell death in melanoma cells [5]. Rapamycin, an mTOR inhibitor, induces autophagic cell death in MG63 osteosarcoma cells [6]. In contrast, autophagy is actively involved in cancer cell resistance to anticancer agents through recycling of cellular energy sources and components. For instance, treatment with chloroquine, an autophagy flux inhibitor, overcomes anti-estrogen resistance of MCF-7 cells [7]. Chloroquine also enhanced a cytotoxicity of temozolomide in glioma cells [8]. Thus, potential role of autophagy in cancer chemotherapy remains elusive.

Lipid-sensing G-protein-coupled receptors (GPCRs) are highly expressed in pancreatic β-cells and implicated with metabolic symdroms [9]. Previous studies examined the diverse functions of lipid-sensing receptors in the development and progression of cancer. GPR120 is activated by ω-3 fatty acids and promotes tumor progression and angiogenesis in prostate cancer [10, 11]. Activations of GPR43 and GPR109A which sense short-chain fatty acids suppress cell proliferation of colon cancer cells and tumorigenesis of breast cancer cells [12, 13]. Oleic acid, an endogenous ligand of GPR40, promotes the proliferation of breast cancer cells, but TAK-875, a synthetic ligand of GPR40, inhibits the tumor growth of melanoma [14, 15]. GPR119 is activated by endogeneous oleoylethanolamine(OEA) and mainly coupled to Gαs signaling [9]. Although GPR119 is a promising target for type II diabetes and fatty liver diseases [16], a role of GPR119 in cancer has not been studied. Here, we found that GPR119 was ubiquitously expressed in human breast cancer cell lines and tumor tissues. We investigated in vitro and in vivo combination effects of GPR119 agonists with TKIs in breast cancer and hepatoma cells, and clarified the mechanistic basis for the anticancer effects of GPR119 agonists, focusing on autophagy inhibition.

## Methods

### Cell culture

Breast cancer cell lines (MCF-7, MDA-MB-231 and TamR-MCF-7) were cultured in Dulbecco’s modified Eagle’s medium (DMEM) containing 10% fetal bovine serum and 1% penicillin-streptomycin. MCF-10A was cultured in media as described previously [17]. Other breast cancer cell lines were cultured in RPMI1640 media. Hepatocellular carcinoma cell lines (HepG2 and HepG2-X cells) were cultured in DMEM containing 10% fetal bovine serum and 1% penicillin-streptomycin.

### Antibodies and reagents

MBX-2982, GSK1292263, gefitinib and sorafenib were obtained from Medchemexpress (Monmouth Junction, NJ, USA). Chloroquine, 3-methyladenine (3-MA) and other reagents were purchased from Sigma-Aldrich (St. Louis, MI). Anti-GPR119 antibody was supplied from Abcam (Cambridge, UK). Anti-monocarboxlyate transporter (MCT) 1, anti-MCT2, anti-MCT4, anti-lactate dehydrogenase (LDH) A, anti-LDHB antibodies were purchased from Santa Cruz Biotechnology (Dallas, TX, USA). Other antibodies including LC3B were supplied from Cell Signaling Technology (Danvers, MA, USA). GFP-LC3B plasmid were kindly donated from Dr. Kim J (University of Florida, Gainesville, FL, USA).

### Incucyte® live cell analysis system for the determination of cell proliferation and caspase-3/7 activity

Cells were seeded in 96 well plate and real-time monitored by Incucyte® system (Essenbio science, Ann Arbor, MI, USA). Sets of images were acquired and analyzed by Incucyte® basic software. Caspase3/7 activity in apoptotic cells was visualized with Kinetic caspase 3/7 reagent (cat.4440, Essenbio science). The half maximal inhibitory concentration (IC_50_) values were calculated through non-linear regression analysis using SigmaPlot ver. 12 (Systat Software Inc., San Jose, California, USA).

### Western blot analysis

Cells were washed with cold phosphate-buffered saline (PBS) and lysed in lysis buffer (150 mM Tris-Cl (pH.7.6), 10% NP-40, protease inhibitors and phosphatase inhibitors) and centrifuged at 16000 *g*, 4 °C. The proteins were separated in sodium dodecyl sulfate (SDS)-polyacrylamide gel electrophoresis (PAGE) and transferred to nitrocellulose membrane. The membranes were incubated with specific primary antibodies diluted in 5% skim milk in Tween 20-containing PBS and corresponding secondary antibodies.

### Reverse transcriptase-polymerase chain reaction (RT-PCR) and quantitative real-time PCR (qPCR)

Total RNA was extracted using Trizol reagent (Invitrogen, Carlsbad, CA, USA). cDNA was synthesized by reverse transcriptase kit (iNtRON, Seoul, South Korea) and PCR was performed using specific primers: human GPR119 F: 5’-CTCCCTCATCATTGCTACTAA-3′, R: 5’-ACAGCCAGATTCAAGGTG-3′. GAPDH F: 5’-AGCCACATCGCTCAGA CAC-3′, R: 5’-GCCCAATACGACCAAATCC-3′. The SYBR Green qPCR amplification was conducted with MiniOpticon real time PCR detection system (Bio-Rad laboratories Inc., Hercules, CA, USA).

### Immunohistochemistry

Forty-nine human breast cancer tissues were obtained from Chungnam National University hospital (IRB approval #: CNUH 2015–03-001). Tissue sections were processed from formalin-fixed paraffin-embedded tissue samples according to previously described method [18]. Tissue sections were stained with anti-GPR119 or anti-estrogen receptor α antibody as primary antibody and images of tissue sections were taken by a bright-field microscopy. In statistics, the pearson’s correlation coefficient between GPR119 and clinical pathology was calculated from sigma plot.

### 1H-NMR analysis for lactate and glucose determination

Cell lysates and media were dried using speed vacuum concentrator and reconstructed in appropriate buffer (2 mM Na_2_HPO_4_, 5 mM NaH_2_PO_4_, 0.025% trimethylsilyl propionate in 99.9% D2O). Proton-NMR (1H-NMR) spectroscopy was performed by Bruker 500 MHz spectrometer (Bruker Coperation, Billerica, MA, USA). Lactate was also determined by lactate assay kit (Abcam, Cambridge, UK). Sample preparation and protocols were performed according to the manufacture’s instruction.

### Xenograft analysis

Female Balb/c-nu mice were purchased from Raonbio (Seoul, South Korea). Animal studies were performed according to the regulation and an approval of Seoul National University Institutional Animal Care and Use Committee (Approval #: SNU-140106-1). Briefly, five-week-old female BALB/c-nude mice were inoculated with cancer cells in flank side. The mice were intraperitonially injected with 17-β-estradiol (2 mg/kg/day). Gefitinib (1 mg/kg/day, 5 times a week) and MBX-2982 (10 mg/kg/day, 5 times a week) were orally administered to the mice for 40 days.

### Transmission Electron microscopy (TEM)

Cells were fixed with karnovskys fixative and washed 3 times with 2 ml 0.05 M cacodylate buffer and post-fixed with 2% osmium tetroxide for 2 h. After washing with distilled water, cells were stained with 0.5% uranyl acetate and dehydrated with a series of ethanol and propylene oxide. Cells were embeded in spurr’s resin and polymerized at 70 °C. The blocks were trimmed to ultrathin section using an ultramicrotome (EM UC7, Leica, Wetzlar, Germany) and observed with Transmission Electron Microscope (JEM1010, JEOL, Tokyo, Japan).

### siRNA transfection and shRNA infection

siGENOME SMARTpools systems for ATG7 (cat # MQ-020112-01-0002) knockdown and scramble (cat # D-001206-13-05) were purchased from Dharmacon (Lafayette, CO). MCF-7 cells and HepG2 cells were transfected with siRNA using FuGENE® HD Transfection Reagent (Promega, Madison, WI, USA). shRNA lentivirus particles for GPR119 (cat # SHCLNV-NM_178471) and Nontarget control (cat # SHV0002) were purchased from Sigma-Aldrich. MCF-7 cell was incubated with lentivirus particles and hexamidine bromide. Stable cell lines were generated by puromycin treatment for more than 2 weeks.

### Mitochondria enrichment fraction isolation

Crude mitochondria were isolated by sucrose gradient purification. Cells were seeded in 150 mm^2^ culture plate and treated with MBX-2982 for 6 h. The cells were washed 3 times with PBS, harvested with trypsin and suspended in 1 ml NKM buffer (1 mM Tris-Cl, pH 7.4, 0.13 M NaCl, 5 mM KCl, 7.5 mM MgCl_2_). The cell mixture was centrifuged and suspended in 0.6 ml homogenization buffer (10 mM Tris-Cl, pH 6.7, 10 mM KCl, 0.15 mM MgCl_2_) and homogenized using a pestle and glass potter. The homogenate was mixed with 0.1 ml 2 M sucrose and centrifuged at 1200 *g* for 5 min and repeated twice. The supernatant was transferred and centrifuged at 7000 *g* for 10 min.

### Mitochondrial function assay

OCR and ECAR were monitored using an XFp analyzer (Seahorse Bioscience, North Billerica, MA, USA) and XFp cell mito-stress test kit (Seahorse Bioscience). 3 × 10^3^ cells were seeded in XFp cell culture miniplate and growth media were replaced with XFp assay media 1 h before the test. All the reagents and assay conditions were followed by manufacturer’s instructions.

### Flow cytometry analysis

For cell cycle analysis, cells were fixed in 70% ethanol overnight, washed twice with PBS and suspended in staining buffer (0.1% Triton X-100, 0.2 mg/ml RNase A, 1 μg/ml Propidium iodide(PI) in PBS) for 10 min. For apoptosis detection, cells were trypsinized, washed with PBS and stained with PI and annexin V in binding buffer (10 mM HEPES, pH 7.4, 140 mM NaCl, 2.5 mM CaCl_2_) for 15 min. The stained cells were washed twice with PBS and analyzed by FACS caliber (BD science, Franklin Lakes, NJ, USA).

### Statistical analyses

Data are presented as mean ± S.D. or S.E. Student’s t-test was used to analyze differences between experimental groups. Values of *p < 0.05 or **p < 0.01 or ***p < 0.005 were considered significant.

## Results

### GPR119 expression in human breast cancer cells and tumor tissues

**Fig. 1:**
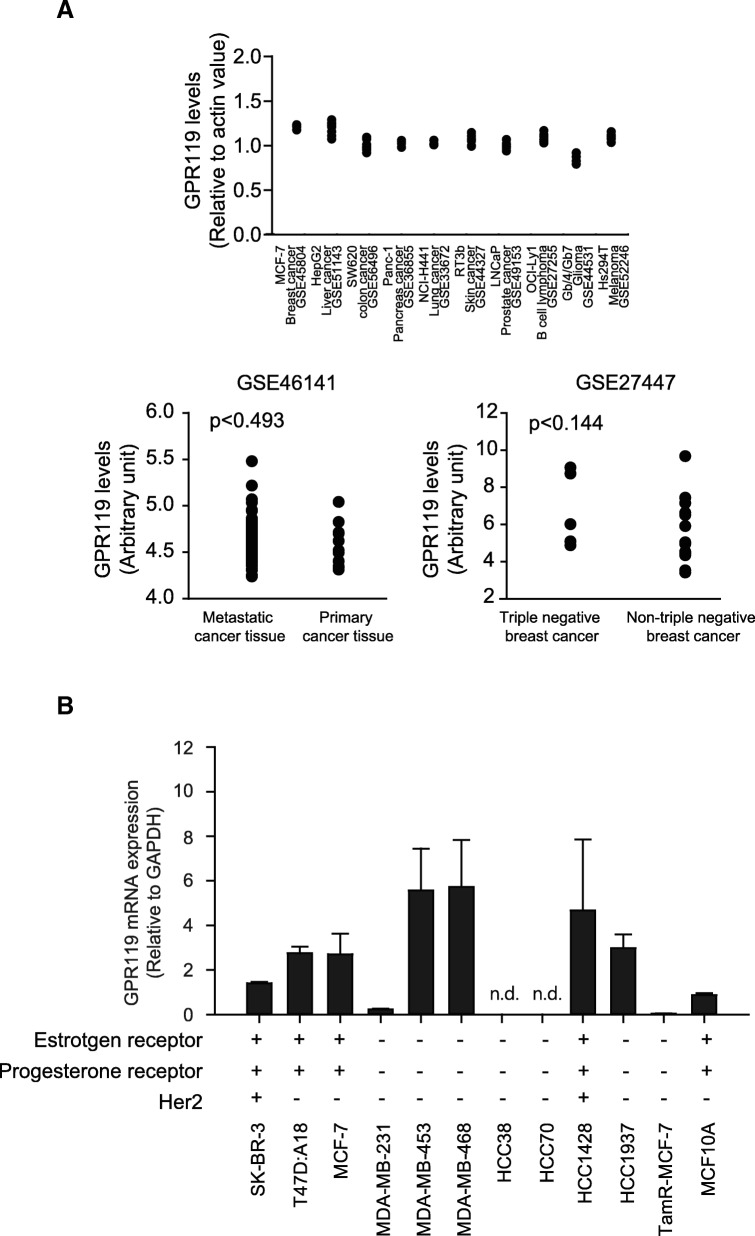
GPR119 expression in human breast cancer cells and tumor tissues. **a** Gene Expression Omnibus profiles of GPR119 in human breast cancer. Upper, relative mRNA levels of GPR119 in human cancer cell lines; lower, GPR119 mRNA expression in metastatic and primary breast cancer tissues; GPR119 mRNA expression in triple negative breast cancer (TNBC) and Non-TNBC tissues. **b** Expression levels of GPR119 mRNA in various human breast cancer cell lines were verified by real-time qPCR. MCF10A cells were used as normal mammary epithelial cells

We found GEO datasets containing GPR119 transcript levels in various cancer cell types (Fig. 1a, upper panel). Although significant differences were not seen between benign and malignant cancer tissues, GPR119 mRNA was identified from human breast cancer tissue datasets (Fig. 1a). By public dataset analysis, GPR119 mRNA was not associated with cancer metastasis or pathology classification of breast cancer (Fig. 1a, lower panels). We further quantified GPR119 mRNA expression in 11 breast cancer cell lines and MCF10A mammary epithelial cell line by real-time qPCR analyses. Compared to MCF-10A, GPR119 mRNA was amplified in seven breast cancer cell lines, and there were no correlation between existence of hormone receptors or Her2 and GPR119 exprtession (Fig. 1b). Immunohistochemistry showed that 19 samples from 49 human breast cancer tissues were GPR119 positive, but there were no remarkable changes in GPR119 expression depending on TMN stages and tumor stages of patients or estrogen receptor (ER)-α (Table 1). Thus, GPR119 was constitutively expressed in human breast cancer.

**Table 1:** Correlation between GPR119 expression and pathological parameters of breast cancer patients

Parameter	GPR119 (IHC)		Pearson correlation (*p*-value)
	Negative	Positive	
T stage			0.535
T1	13	8	
T2	17	11	
T3	0	0	
N stage			0.892
N0	18	10	
N1	9	8	
N2	3	1	
M stage			No value
M0	30	19	
M1	0	0	
Tumor stage			0.896
1	9	5	
2	18	13	
3	3	1	
ER alpha (IHC)			0.0898
Negative	9	4	
Positive	21	15	

### Proliferation inhibition and intrinsic apoptosis by GPR119 agonist in breast cancer cells

**Fig. 2:**
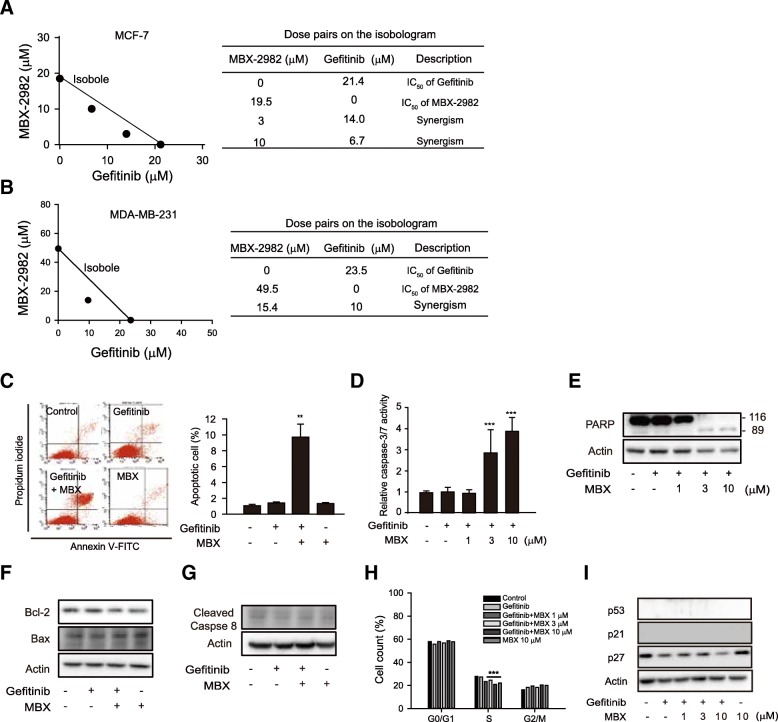
Enhanced proliferation inhibition by GPR119 agonists in breast cancer cells. **a** and **b** Synergistic effects of GPR119 agonist on cell proliferation inhibition by gefitinib in MCF-7 **a** and MDA-MB-231 cells **b**. Isobole model was used to evaluate drug combination effect. **c** Apoptosis induction by gefitinib with MBX-2982. Apoptosis was determined by PI and annexin V staining. The stained cells were analyzed by flow cytometry in MCF-7 cells. Annexin V-positive pro-apoptotic and late-apoptotic cells were counted by BD CellQuest Pro software. **d** Caspase3/7 activity. MCF-7 cells were preincubated with DEVD-NucViewTM 488, and exposed to gefitinib and MBX-2982 for 60 h, and caspase-3/7-selective green fluorescence was monitored. Green fluorescence intensity was calculated by Incucyte® ZOOM basic analyzer. **e** PARP cleavage by gefitinib with MBX-2982. MCF-7 cells were incubated in the presence of gefitinib or gefitinib with MBX-2982 for 48 h. Western blot analyses were performed to determine PARP cleavage. **f** Expression of Bcl-2 and Bax. **g** Expression of cleaved caspase-8 (active form). MCF-7 cells were incubated with 10 μM gefitinib, 10 μM MBX or gefitinib/MBX for 24 **h**. Cell cycle analysis h and cell cycle-associated protein expression **i**. MCF-7 cells were treated with MBX-2982 (MBX) for 24 h and fixed with ethanol. Cell cycle was analyzed by propidium iodide (PI) staining in MCF-7 cells. For the quantification of protein expression of p53, p21 and p27, MCF-7 cells were incubated with 1-10 μM MBX-2982 for 24 h. Data represent the mean ± S.D. (n=3). ** *p* < 0.01, *** *p* < 0.005 significant difference versus control group

Aberrant EGFR signaling is involved in poor prognosis of many types of cancer. One of the current treatment options for breast cancer is EGFR-TKI as well as anti-hormone agents [19]. However, clinical outcomes of EGFR-TKI are less successful than expected [20]. Hence, we assessed whether GPR119 agonist provides synergistic benefit with EGFR-TKI in human breast cancer. To assess the potential synergism of two drugs, ‘isobole model’ has been frequently used [21]. In the isobole model, the position of coordinates of drug doses displayed the effect of combination (additive, synergistic, antagonistic effect). The IC_50_ values of gefitinib and MBX-22982 in cell proliferation of MCF-7 cells (a representative ER+ breast cancer cell line) were 21.2 μM and 18.8 μM, respectively. The dose pairs of the combination of gefitinib and MBX-2982 were below the isobole indicating the synergistic effect of the drug combination (Fig. 2a). We also calculated the isobologram of the drug combination on MDA-MB-231 cells [a triple negative breast cancer (TNBC) cell line] and observed the same results (Fig. 2b). Another GPR119 agonist used in clinical trials, GSK1292263, also potentiated the anti-proliferative effect of gefitinib on MCF-7 and MDA-MB-231 cells (Additional file 1: Figure S1A and B). We further tested the combined effects of GPR119 agonist with gefitinib on cell proliferation of MDA-MB-468 (a TNBC cell line) and SK-BR-3 cells (a Her2+ and ER+ breast cancer cell line). Cotreatment with 3 μM MBX-2982 and 100 nM gefitinib additively suppressed cell proliferation of MDA-MB-468 or SK-BR-3 cells (Additional file 1: Figure S1C). The data suggest that GPR119 aognist with gefitinib synergistically inhibits proliferation of breast cancer cells.

We then assessed the effect of OEA (endogenous GPR119 ligand) on proliferation of MCF-7 cells. Because EC_50_ values of MBX-2982 and OEA for GPR119 are 3.9 nM and 0.2–5 μM, respectively [22], pharmacological potency of MBX-2982 is 51.3–1282.1 fold higher than OEA. When we assessed cell proliferation inhibitory effect of OEA (10 mM) in MCF-7 cells, the compound did not change the basal cell growth (Additional file 1: Figure S1D). However, co-treatment with OEA and gefitinib significantly reduced cell proliferation of MCF-7 cells compared to gefitinib alone group (Additional file 1: Figure S1D).

To examine a possible mechanism for the anti-cancer effects of GPR119 agonists, flow cytometry analyses were performed after exposure of MCF-7 cells to MBX-2982 for 48 h. Annexin V and propidium iodide (PI) staining revealed that a late-apoptotic population was 6.9-fold enhanced in a MBX-2982-gefitinib cotreated group compared to the gefitinib-alone group (Fig. 2c). Representative apoptosis indices, caspase3/7 activity and poly (ADP-ribose) polymerase (PARP) cleavage also increased with cotreatment for 36 h (Fig. 2d and e). The relative ratio of Bcl-2/Bax expresion represents intrinsic apoptosis marker, and caspase-8 activation is related with extrinsic apoptosis pathway [23]. Although Bax expression was not altered, Bcl-2 expression was decreased by cotreatment with MBX-2982/gefitinib (Fig. 2f). Changes in cleaved caspase-8 (active form) were not observed in all treatment groups (Fig. 2g). We further analyzed cell cycle progression and the expression of cell cycle marker proteins. Cell population percentage of S phase was significantly reduced by co-treatment with gefitinib and MBX-2982, and p27 expression was also remarkably suppressed (Fig. 2h and i). These results indicate that the anti-proliferative effect of GPR119 agonist seemed to be related with impairment of cell cycle progression as well as stimulation of late apoptosis.

### Inhibition of EGFR-TKI-induced autophagy by MBX-2982 in breast cancer cells

**Fig. 3:**
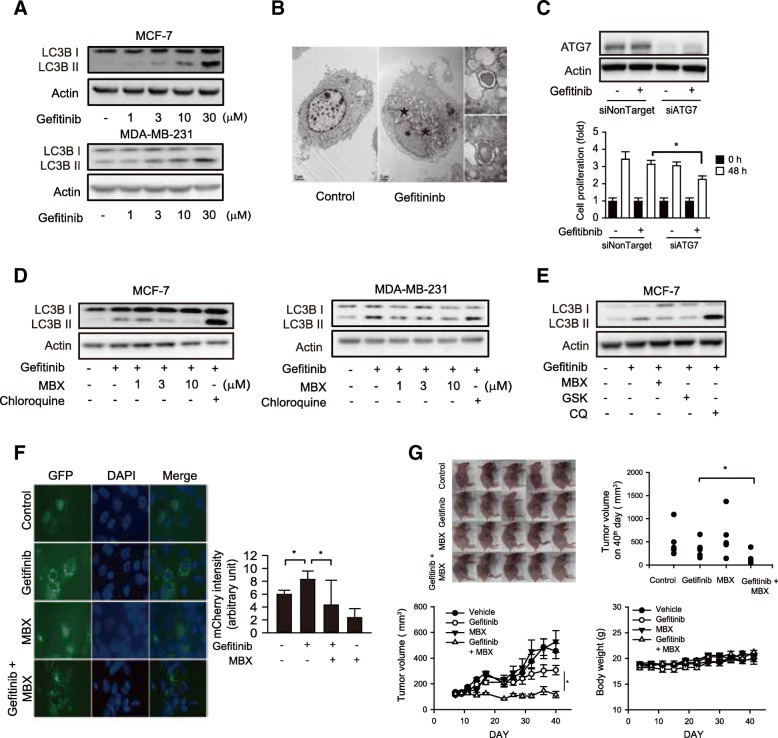
Inhibition of gefitinib-induced autophagy by GPR119 ligands in breast cancer cells. **a** Autophagy induction by gefitinib in human breast cancer cells. LC3B I/II were measured by immunoblottings in breast cancer cells (MCF-7 and MDA-MB-231 cells). Cells were incubated with 1-30 μM gefitinib for 24 h. **b** Autophagosome formations in gefitinib-treated MCF-7. Autophagosome formation was visualized by TEM in MCF-7 cells. Cells were incubated with 10 μM gefitinib for 24 h. Star marks indicate double lipid layer vesicle structures. **c** Effect of ATG7 siRNA on anti-proliferative effect of gefitinib. ATG7 expression was detected by western blotting after siATG7 transfection (upper) and cell proliferation was monitored by Incucyte® ZOOM basic analyzer in MCF-7 cells (lower). Data represent the mean ± S.D. (n=6). **d** Inhibition of gefitinib-induced autophagy formation by MBX-2982 (MBX). LC3B I/II were measured by western blottings in breast cancer cells incubated with 10 μM gefitinib in the presence or absence of 1-10 μM MBX or 3 μM chloroquine. **e** Inhibition of gefitinib-induced autophagy formation by GSK1292263 (GSK). LC3B I/II were measured by western blottings in MCF-7 cells incubated with 10 μM gefitinib, 10 μM MBX, 10 μM GSK or 3 μM chloroquine for 24 h. **f** Inhibition of autophagosome puncture formation by MBX. Green fluorescence puncta were detected by fluorescence microscopy after LC3-GFP transfection in MCF-7 cells (left). Red fluorescence was calculated in MCF-7 cells after mCherry-GFP-LC3 plasmid transfection by Incucyte® ZOOM basic analyzer (right). MCF-7 cells were incubated with 10 μM gefitinib in the presence or absence of 10 μM MBX. Data represent the mean ± S.D. (n=3). **g** Tumor growth of MCF-7 xenograft was monitored for 40 days. Data represent the mean ± S.E. (n=5). * *p* < 0.05, significant difference between the indicated two groups

Autophagy process triggered by autophagosome formation shows dual functions; cell survival and cell death. Chemotherapies including EGFR-TKI induce functional autophagy in diverse cancer cells types [24]. To confirm if gefitinib induces autophagy in breast cancer cells, we determined LC3B II expression as a marker of autophagosome formation [25]. LC3B II protein increased with gefitinib treatment in MCF-7 and MDA-MB-231 cells (Fig. 3a). Transmission electron microscopy (TEM) showed that a lipid bilayer structure in the cytoplasm (autophagosomes) formed in MCF-7 cells with gefitinib treatment (Fig. 3b). When ATG7 was silenced by siRNA transfection to block autophagy, gefitinib-induced inhibition of cell proliferation was potentiated (Fig. 3c), suggesting that gefitinib-induced autophagy is a survival mechanism of cancer cells.

Gefitinib-induced LC3B II expression was suppressed in MCF-7 and MDA-MB-231 cells by MBX-2982 (Fig. 3d). GSK1292263 also inhibited conversion to LC3B II in response to gefitinib (Fig. 3e). Chloroquine, a blocker of autophagolysosome formation, accumulates autophagosomes in cells [25]. We confirmed these results using MCF-7 cells transfected with a green fluorescence protein (GFP)-tagged LC3B overexpression plasmid. GFP fluorescence was ubiquitous in whole cells after vehicle treatment, while green punctuate structures were observed in gefitinib-treated MCF-7 cells, and the number of GFP puncta was diminished by MBX-2982 treatment (Fig. 3f, left). In LC3B puncta formation assays with red fluorescence protein (mCherry) tagging, the number of puncta significantly decreased with MBX-2982 treatment (Fig. 3f, right). We then tested the effects of MBX-2982 on tumor growth of xenograft implants of MCF-7 cells. Because MCF-7 cells show hormone-dependent growth, 17-β-estrogen (2 mg/kg/day) was subcutaneously injected into tumor-bearing mice. Tumor growth was moderately reduced by oral administration of gefitinib, whereas tumor growth was completely suppressed by co-administration of gefitinib with MBX-2982 (Fig. 3g). The data demonstrate that GPR119 agonist inhibits autophagosome formation in cancer cells treated with EGFR-TKI and possess anticancer effects against breast cancer.

### Cell proliferation inhibition by GPR119 agonist and target chemotherapies

**Fig. 4:**
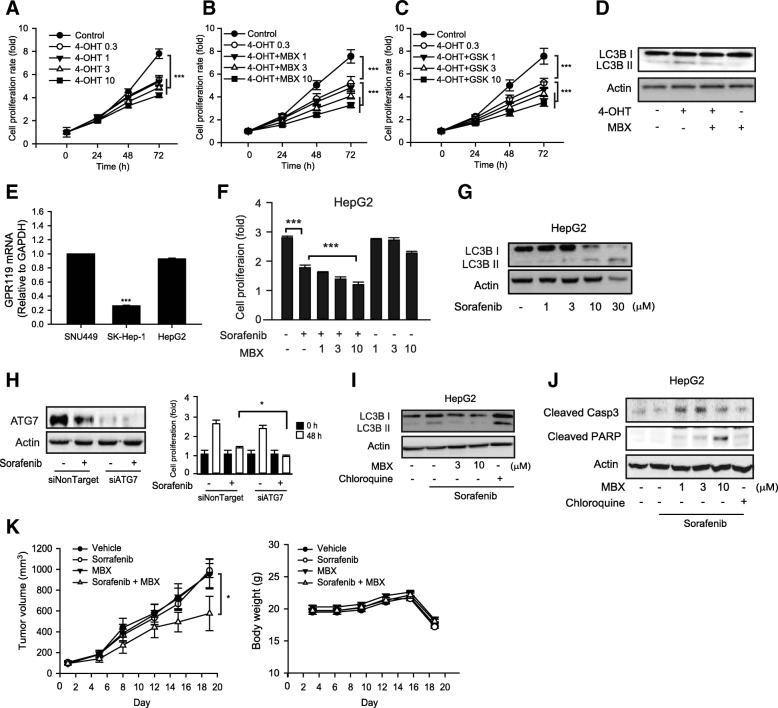
Enhancement of target therapy-induced proliferation inhibition by GPR119 agonists. **a** Cell proliferation was monitored after treatments with vehicle or 4-OHT (0.3-10 μM). **b** and **c** Cell proliferation was monitored after treatments with 0.3 μM 4-OHT and GPR119 agonists (1-10 μM). Data represent the mean ± S.D. (n=6). **d** LC3B I/II were measured by western blottings in breast cancer cells incubated with 0.3 μM 4-OHT in the presence or absence of 10 μM MBX for 24 h. **e** Expression levels of GPR119 mRNA in three human hepatocellular carcinoma cell lines were verified by real-time qPCR. **f** HepG2 cells were incubated with 10 μM sorafenib or MBX (1-10 μM) for 48 h, and cell proliferation was monitored by MTT assays. Data represent the mean ± S.D. (n=6). **g** Autophagy induction by sorafenib in HepG2 cells. The cells were incubated with vehicle or sorafenib (1-30 μM) for 18 h. **h** ATG7 expression was detected by western blotting after siATG7 transfection (left) and cell proliferation was monitored by Incucyte® ZOOM basic analyzer in HepG2 cells (right). Data represent the mean ± S.D. (n=6). **i** LC3B I/II were measured by western blottings in HepG2 cells incubated with 10 μM sorafemib in the presence or absence of MBX (3 and 10 μM) or 3 μM chloroquine for 18 h. **j** Cells were incubated with sorafenib in the presence or absence of MBX (1-10 μM) for 24 h. **k** In vivo effect of MBX-2982 on tumor growth of HepG2-X xenograft. Sorafenib (10 mg/kg), MBX-2982 (10 mg/kg) or Sorafenib with MBX-2982 were orally administered (5 times a week) and tumor growth was monitored for 18 days. Data represent the mean ± S.E. (n=6). * *p* < 0.05, *** *p* < 0.005, significant difference between the two indicated groups

We further tested synergistic effects of GPR119 agonist with other target therapeuctic agents, tamoxifen and sorafenib. Tamoxifen is a selective estrogen receptor modulator and is widely used to treat or prevent estrogen receptor-positive breast cancer [26]. Cell proliferation of MCF-7 cells was partially inhibited by 0.3–10 μM 4-hydroxytamoxifen (4-OHT), an active metabolite of tamoxifen, but concentration-dependency was not observed (Fig. 4a). Whereas, combination of 0.3 μM 4-OHT with MBX-2982 or GSK1292263 additively inhibited cell proliferation of MCF-7 cells (Fig. 4b and c). Moreover, 4-OHT-induced LC3B II expression was reversed by MBX-2982 co-treatment (Fig. 4d).

Sorafenib as a multi-kinase inhibitor is now used for the treatment of hepatocellular carcinoma [27]. When we determined GPR119 mRNA expression in HepG2, SNU-449 and SK-Hep1 hepatocellular carcinoma cell lines, GPR119 mRNA was similarly detected in HepG2 and SNU-449 cells, but its expression level was relatively low in SK-Hep1 cells (Fig. 4e). We also found that cell proliferation of HepG2 cells, a human hepatoma cell line was additively inhibited by the combination of sorafenib with MBX-2982 (Fig. 4f). In a similar pattern to MCF-7 cells treated with gefitinib, LC3B II expression was increased in HepG2 cells exposed to sorafenib (Fig. 4g), and inhibitory effect of sorafenib on cell proliferation was potentiated by ATG7 siRNA transfection (Fig. 4h). Moreover, sorafenib-induced LC3B II expression was inhibited by MBX-2982 in HepG2 cells (Fig. 4i), and apoptotic markers such as cleaved forms of caspase-3 and PARP were enhanced by cotreatment with sorafenib and MBX-2982 in HepG2 cells (Fig. 4j). We also tested the in vivo effects of MBX-2982 on xenograft implants of HepG2-X cells, a HepG2 subclone for xenograft. Co-administration of MBX-2982 with sorafenib synergistically inhibited tumor growth of HepG2-X cells (Fig. 4k). These results indicate that cancer cells induce autophagy in response to target therapies as a survival mechanism and GPR119 agonist possesses anticancer effects via autophagy inhibition.

### GPR119 signaling in autophagy inhibition by MBX-2982

**Fig. 5:**
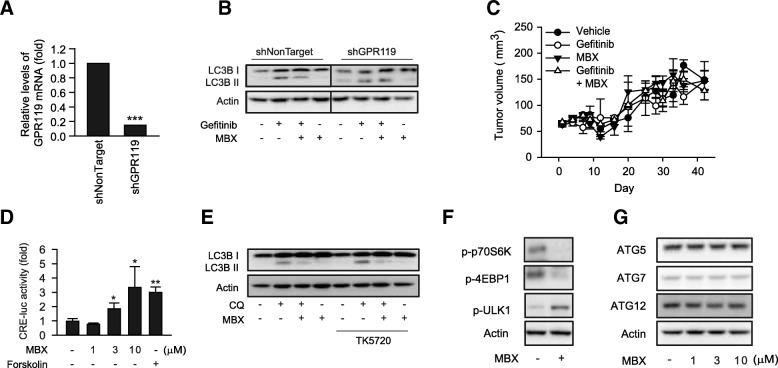
GPR119 signaling in autophagy inhibition by MBX-2982. **a** GPR119 mRNA expression in GPR119 shRNA-infected cells. MCF-7 cells were infected with shGPR119 or shNonTarget lentivirus particle, and GPR119 mRNA expression was determined by real-time qPCR. Data represent the mean ± S.D. (*n* = 3)(*** *p* < 0.005, significant difference versus shNonTarget-infected control). **b** Reversal of MBX-2982 (MBX)-induced autophagy inhibition in GPR119 knock-down cells. LC3B I/II were measured by western blotting in shGPR119 or shNonTarget-infected MCF-7 cells. **c** Xenograft analysis. Tumor growth of shGPR119-MCF-7 xenograft was monitored for 41 days. Balb/c-nu mice were orally administered with gefitinib (1 mg/kg), MBX (10 mg/kg) or gefitinib with MBX (5 times a week). Data represent the mean ± S.E. (*n* = 3). **d** CRE reporter activity. MCF-7 cells were transiently transfected with CRE-luciferase reporter plasmid (CRE-luc) and its reporter activity was measured using luminometer. 10 μM forskolin, an adenylyl cyclase activator, was used as positive control. Data represent the mean ± S.D. (*n* = 3)(* *p* < 0.05, ** *p* < 0.01, significant difference versus control group). **e** Effect of PKA inhibitor on autophagy inhibition by MBX. MCF-7 cells were preincubated with 10 μM of TK5720, a PKA inhibitor for 30 min and then treated with 3 μM chloroquine in the presence or absence of 10 μM MBX. **f** Effect of MBX on mTOR signaling pathway. mTOR signaling pathway was estimated by immunoblottings for phosphorylated p70S6 kinase, phosphorylated 4EBP1 and phosphorylated ULK1 in MCF-7 cells incubated with 10 μM MBX-2982 for 24 h. **g** Effects of MBX on the protein levels of ATG5, ATG7 and ATG12. MCF-7 cells were incubated with MBX (1–10 μM) for 24 h

We established GPR119 knockdown cells by shRNA infection to assess whether autophagy inhibition by MBX-2982 was due to target receptor-mediated response. MCF-7 cells were infected with shGPR119 lentivirus and puromycin-resistant GPR119 knockout clones of MCF-7 cells were selected. GPR119 mRNA was determined by quantitative RT-PCR (Fig. 5a). MBX-2982 inhibited gefitinib-induced LC3B II protein expression in nontargeted shRNA-infected MCF-7 cells. GPR119 knockdown reversed the inhibition of LC3B II expression by MBX-2982 (Fig. 5b), implying that GPR119 signaling was required for the inhibitory effect of MBX-2982. Moreover, the coadministration of gefitinib with MBX-2982 did not reduce the tumor volume in xenografts implanted with shGPR119-MCF-7 cells (Fig 5c), which imply that GPR119 signaling is required for anti-cancer effect of MBX-2982.

Stimulation of GPR119 coupled to Gαs results in cAMP increase and protein kinase A (PKA) activation [28]. When we determined reporter activity using a cAMP response element (CRE) to represent intracellular cAMP production, 10 μM MBX-2982 increased the luciferase reporter activity (Fig. 5d). However, cAMP pathway inhibition with TK5720, a specific PKA inhibitor, did not restore the diminished LC3B II expression by MBX-2982 in MCF-7 cells (Fig. 5e). Because the mTORC1 pathway suppresses ULK1 which initiates autophagosome formation, we hypothesized that mTORC1 was activated or ULK1 was inactivated by MBX-2982. However, downstream targets of mTORC1, phosphorylation of ribosomal protein S6 kinase β-1 (p70S6K) and eukaryotic translation initiation factor 4E-binding protein 1 (4EBP1), were inhibited by MBX-2982 (Fig. 5f). In addition, ULK1 phosphorylation increased at 24 h exposure of MCF-7 cells to MBX-2982 (Fig. 5f). Protein components that elongate autophagosomes, ATG5, ATG7 and ATG12 were also not significantly affected by MBX-2982 (Fig. 5g). The data suggest that autophagy inhibition by GPR119 agonist is not mediated through classical GPR119 signaling or the PI3K/mTORC1/ULK1 pathway.

### Metabolic shift by MBX-2982 and inhibition of gefitinib-induced autophagy by lactate

**Fig. 6:**
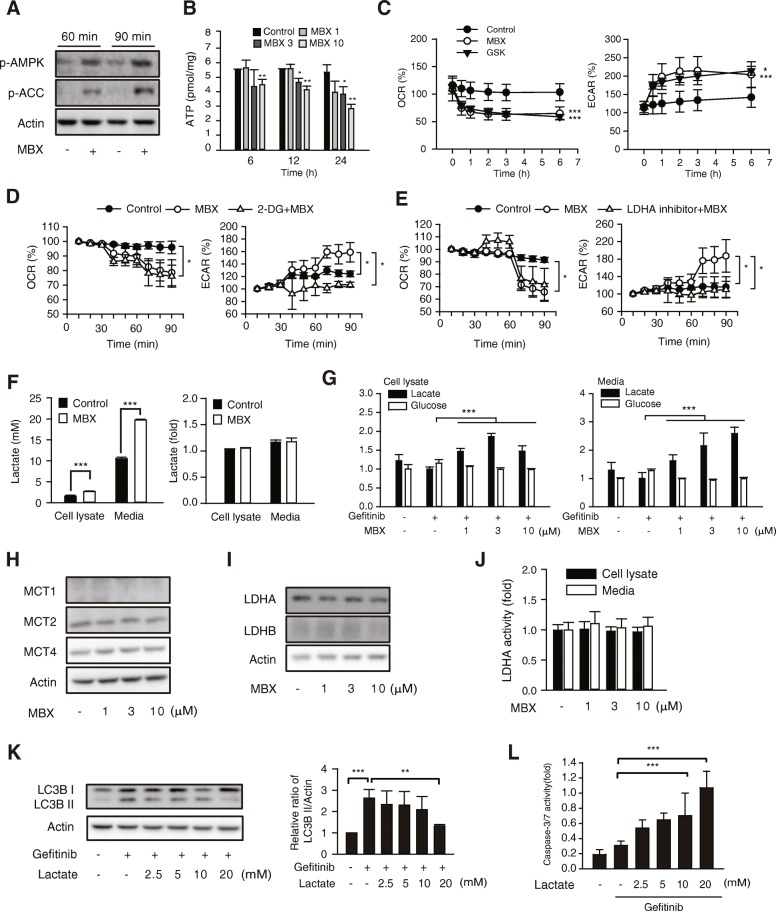
Metabolic shift by MBX-2982 and inhibition of gefitinib-induced autophagy by lactate. **a** AMPK activity was determined by immunoblottings for phosphorylated AMPK and phosphorylated ACC proteins in MCF-7 cells treated with 10 μM MBX-2982. **b** ATP content was determined by ATP assay kit in MCF-7 cells. Cells were treated with MBX-2982 (1-10 μM). **c** Effects of GPR119 agonists on mitochondrial OXPHOS and glycolysis in MCF-7 cells. **d** MCF-7 cells were pretreated with or without 2-deoxyglucose (2-DG, 50 mM) for 30 min and incubated with 10 μM MBX. **e** MCF-7 cells were pretreated with or without GSK2837808 (LDH inhibitor, 10 μM) for 30 min and incubated with 10 μM MBX. **f** MBX-induced lactate production in MCF-7 (left) and shGPR119-MCF-7 cells (right). Lactate concentration was assessed by 1H-NMR or lactate assay kit in total cell lysates and culture media. **g** MCF-7 cells were incubated with 10 μM gefitinib in the presence or absence of MBX for 24 h and concentrations of glucose and lactate were determined by 1H-NMR in total cell lysates (left) and culture media (right). **h** and **i** MCF-7 cells were treated with MBX for 24 h and protein expression of lactate transporters and lactate converting enzymes were measured by western blottings. **j** MCF-7 cells were treated with MBX-2982 for 24 h and LDHA activity was determined by LDH assay kit. **k** MCF-7 cells were incubated with 10 μM gefitinib in the presence or absence of lactate (2.5-20 mM) for 24 h, and LC3B I/II were measured by immunoblottings. **l** Caspase3/7 activation by gefitinib with lactate. MCF-7 cells were treated gefitinib (10 μM) with or without lactate (5-20 mM) for 72 h. Data represent the mean ± S.D. (n=3). * *p* < 0.05, ** *p* < 0.01,*** *p* < 0.005 significanct difference between the two indicated groups

We previously reported that MBX-2982 activated AMP-activated protein kinase (AMPK), and pleiotrophic roles of AMPK have been investigated in many types of cancer [22, 29]. Hence, we further investigated a potential role of AMPK in autophagy inhibition by GPR119 agonist. We found that AMPK was activated in MCF-7 cells by MBX-2982, as evidenced by phosphorylation of AMPK or acetyl CoA carboxylase (ACC), a downstream target of AMPK (Fig. 6a), but the activation of upstream kinases such as liver kinase B1 (LKB1) and transforming growth factor-β-activating kinase 1 (TAK1), was not observed (data not shown). On the other hand, cellular ATP production was sustainedly dimished by MBX-2982 in MCF-7 cells (Fig. 6b). Because ATP production mainly relies on mitochondrial oxidative phosphorylation (OXPHOS) [29], we hypothesized that mitochondrial function might be defective in MBX-2982 treated cells. Mitostress assays revealed that exposure of MCF-7 cells to MBX-2982 or GSK1292263 for 30 min, 2 or 6 h continuously suppressed oxygen consumption rate (OCR), which reflects ATP production capacity in mitochondria (Fig. 6c, left). Elevated extracellular acidification rate (ECAR) indicates that energy metabolism inclines toward glycolysis. Both the GPR119 agonists increased ECAR in MCF-7 cells (Fig. 6c, right). We then assessed the effects of 2-deoxlyglucose (2-DG), a competitive inhibitor of glycolysis. 2-DG restored the elevated ECAR without OCR change in MBX-2982-treated MCF-7 cells (Fig. 6d). Lactate is produced by lactate dehydrogenase A (LDHA) during the process of glycolysis. GSK2387808, a LDH inhibitor, also suppressed MBX-2982-mediated increases in ECAR without changing OCR (Fig. 6e). These results domonstrate that GPR119 agonsits cause a metabolic shift toward glycolysis in MCF-7 cells.

To further investigate the role of enhanced glycolysis by GPR119 agonists, we determined intracellular and extracellular levels of lactate, an end product of glycolysis. Extracellular and intracellular levels of lactate were ~ 1.8-fold increased by MBX-2982, and lactate in media even reached 20 mM (Fig. 6f, left). In shGPR119-MCF-7 cells, increased lactate by MBX-2982 was not detected (Fig. 6f, right). We also found that lactate levels in either cell lysates or culture media also increased by MBX-2982 in MCF-7 cells treated with gefitnib, while intracellular amd extracellular glucose contents were slightly decreased (Fig. 6g). To clarify the effects of MBX-2982 in cellular pathways of lactate production, protein levels for MCT1/2, influx transporters of lactate and MCT4, an efflux transporter of lactate, were quantified. Expression of the transporters did not change with MBX-2982 (Fig. 6h). LDHA protein and enzyme activity were not altered by MBX-2982 (Fig. 6i and j). LDHB, which catalyzes conversion of lactate to pyruvate, was not detected in MCF-7 cells (Fig. 6i).

We then tested whether lactate production is involved in autophagy inhibition by MBX-2982. Given the detected concentration of lactate in culture media (Fig. 6f), 2.5–20 mM lactate was cotreated with gefitinib in MCF-7 cells. Interestingly, the LC3B II expression induced by geifinib was inhibited by 20 mM lactate (Fig. 6k). We further ascertained that co-incubation of MCF-7 cells with 10–20 mM lactate and gefitinib synergistically increased caspase-3/7 activity (Fig 6l). Thus, GPR119 agonist-mediated lactate production may lead to autophagy inhibition and potentiated cancer cell apoptosis with EGFR-TKI.

## Discussion

Chemotherapy resistance frequently occurs in cancer patients and thereby restricts the clinical benefit of the chemotherapeutic agents. The acquired resistance could be cured by the combination with other drugs that discard the resistance mechanism. For example, colorectal cancer harboring KRAS mutation shows the resistance to cetuximab, an EGFR monoclonal antibody. Mitogen-activated protein kinase kinase (MEK) inhibitor could increase the effect of cetuximab through blocking Ras-Raf pathway [30]. Because fulvestrant (pure estrogen receptor antagonist)-resistant breast cancer frequently shows the upregulation of mTOR pathway, the combination of fulvestrant and mTOR inhibitor is more efficious than fulvestrant alone [31]. Autophagy progression is known to be associated with cancer cell survival mechanism in the target therapies [32]. In fact, autophagy inhibitors such as chloroquine and bafilomycin A are cytotoxic to cancer cells [33], and several ongoing clinical trials are using chloroquine alone or the combination of chloroquine with taxane in breast cancer patients (ClinicalTrials.gov Identifier: NCT02333890 and NCT01446016).

GPR119 activation stimulates insulin and GLP-1 secretion in the pancreas and gastrointestinal tract and both the activities are related to Gαs protein-dependent cAMP secretion [34]. In addition, GPR119 expression is upregulated by oxidized low-density lipoprotein in THP-1 human monocyte cell line, and GPR119 overexpression inhibits the development of atherosclerosis in apoE-null mice [35]. It has been shown that some GPCRs are involved in the regulation of autophagy, thus the ligand of these GPCRs are possible to use for autophagy-related diseases [36]. We observed that GPR119 expressed in human breast cancer cell lines and tumor tissues, and GPR119 agonists with gefitinib additively suppressed the growth of breast cancer cells and induced intrinsic apoptosis. Moreover, combination of GPR119 agonist with 4-hydroxytamoxifen in MCF-7 cells as well as with sorafenib in HepG2 cells enhanced the anti-cancer effect of each target therapy. Although GPR119 agonist did not affect cell cycle progression in MCF-7 cells, EGFR TKI-mediated autophagy stimulation was reversed by MBX-2982 treatment. Thus, the anti-autophagy effect of GPR119 ligand may not be specific for cancer cell types or TKI classification.

Here, we also found that mTORC1 signaling was suppressed by GPR119 agonist, which is evidenced by decreased phosphorylations of p70S6K and 4EBP1 by MBX-2982 in MCF-7 cells. Because the activation of p70S6K and 4EBP1 is important for mRNA translation [37], GPR119 agonsit-induced mTORC1 inhibition may lead to the depletion of protein synthesis in cancer cells. Moreover, autophagy supplies recycling nutrients such as amino acids, nucleotides and fatty acids through the degradation of intracellular substrates [38]. Metabolites analysis using LC/Ms./Ms. showed that many animo acids as well as nucleotides were dimished by MBX-2982 treatment in MCF-7 cells (Additional file 2: Figure S2). These results support a notion that GPR119 stimulation in cancer cells results in the deficiency of building blocks for proteins.

Apoptosis induction and autophagy inhibition by a GPR119 agonist might be related to changes in cancer cell metabolism instead of canonical signaling pathway(s) of GRP119. Inhibition of autophagy by MBX-2982 was not restored by cAMP/PKA inhibitor. Instead, our data demonstrated that MBX-2982 suppressed mitochondrial OXPHOS with increased lactate production by glycolysis stimulation. Although future study is needed on why reduced OXPHOS and enhanced glycolysis occur at the same time, intracellular ATP production was eventually decreased by MBX-2982. We suggest that increased lactate production is the cause of autophagy inhibition by MBX-2982. The molecular mechanism for the suppression of autophagosome formation by lactate remains unclear, but may be associated with intracellular pH changes. Although autophagolysosome activity is likely to be amplified by physiologically acidic pH (< 6.4) [39], GPR119 agonist-induced supraphysiological lactate production could suppress autophagosome formation and eventually induce cancer cell death. Intracellular pH regulation via MCTs or LDHs could be important for cancer cell survival. Indeed, inhibitors of MCTs or LDHs are a potential therapeutic target in cancer [40]. Another possibility is that specific lactate receptor(s) may be involved in autophagosome suppression by lactic acid.

GPR81 is activated by extracellular lactate [41–44]. GPR81 is involved in the lipolysis inhibition in adipocytes and in the anti-inflammation in macrophages [41, 42]. Moreover, GPR81 is upregulated in malignant cancers and involved in cell migration and metastasis [44]. Shen et al. have shown that extracellular lactate induces caspase-3-dependent apoptosis through Bax upregulation in GPR81-transfected N2A (mouse neuroblastoma) cells [43]. However, when GPR81 was depleted in MCF-7 cells (Additional file 3: Figure S3A), MBX-2982-mediated autophagy inhibition was not altered (Additional file 3: Figure S3B), indicating that GPR81 may not be associated with lactate-induced autophagy inhibition. From the view of tumor microenvironment, infiltrated macrophages and associated stromal cells promote tumor progression [45]. Tumor-associated macrophages exacerbate tumor progression via the secretion of several cytokines such as interleukin-1β and interleukin-6 [45, 46]. GPR119 agonist-mediated lactate production could suppress the inflammatory immune cells producing tumor-promoting cytokines via GPR81 activation. In support of this notion, robust interleukin-1β and interleukin-6 production were observed in macrophage from GPR81-null mice [41]. The Yeom research group first identified N-myc downstream-regulated gene 3 protein (NDRG3) as a novel binding partner of intracellular lactate [47]. NDRG3 protein bound to lactate is upregulated by enhanced protein stability and accelerates cell growth and angiogenesis of cancer cells by activating the Raf-extracellular signal-regulated kinase pathway [47]. However, NDRG3 protein expression was not detected in MCF-7 cells exposed to MBX-2982 (Additional file 3: Figure S3C). Hence, autophagy inhibition by GPR119 agonist may not be associated with GPR81-dependent signaling by extracellular lactate or NDRG3-dependent signaling by intracellular lactate.

Because a previous study has reported that increased glycolysis and lactate production triggers breast cancer cell stemness and tumor growth in MCF-7 cells [48], GPR119 activation could be related with cancer cell stemness. To assess whether GPR119 agonist induces cancer cell stemness in MCF-7 cells, we performed spheroid formation assay in ultra-low attachment (ULA) plate condition. 10^3^ MCF-7 cells cultured on ULA plate were incubated with vehicle or GPR119 agonist for 96 h. Interestingly, the spheroid volume was significantly diminished by MBX-2982 (Additional file 3: Figure S3D), which suggest that GPR119 agonist does not induce cancer cell stemness, rather inhibits spheroid formation. Martinez-Outschoorn et al. also described that metformin exerts its anti-cancer effects via inducing aerobic glycolysis that has been proposed as a cause of cancer [48]. Hence, the revaluation of glycolysis and lactate production on the cancer cell stemness would be required.

The inhibition of mitochondrial OXPHOS by MBX-2982 appeared to be partially related to a direct action of GPR119 agonist on mitochondria. When we examined mitochondrial stress in GPR119-knockdown MCF-7 cells, GRP119 receptor depletion significantly restored mitochondrial stress (OCR decrease) induced by 3 μM MBX-2982. However, OCR decrease was not restored in GPR119 knockdown cells incubated with 10 μM MBX-2982 (Additional file 4: Fig. S4A). Considering our finding that high concentration of MBX-2982 inhibited OCR in receptor-independent manner, we tested the direct effect of MBX-2982 on mitochondrial complex activity. We found that 10^− 10^–10^− 6^ M MBX-2982 marginally inhibited the enzyme activity of complex I in isolated mitochondria (Additional file 4: Figure S4B). After 3 h incubation of MCF-7 cells with 10 μM MBX-2982, approximately 10% of MBX-2982 was detected in a mitochondrial fraction compared to the amount in whole cell lysates, using LC/Ms./Ms. analysis (Additional file 4: Figure S4C). Hence, a MBX-2982-induced metabolic shift in cancer cells may result from a direct action on mitochondria as well as GPR119 receptor-dependent activity. It has been suggested that several mitochondrial targeting agents could be adopted for cancer chemotherapies. CPI-613, an α-linolic acid derivative, selectively targets altered mitochondrial function and induces cell death in H460 lung cancer cells [49]. Metformin, an AMPK activator, inhibits complex I and is used to treat diverse types of cancer in clinical trials [50].

## Conclusion

GPR119 agonists reduced mitochondrial OXPHOS and stimulated glycolysis in breast cancer cells, with consequent overproduction of lactate that inhibited autophagosome formation. Because autophagy is crucial for the survival of cancer cells exposed to TKIs, GPR119 agonists potentiated the anticancer effects of TKIs. Our findings indicate that the combination of GPR119 agonist with TKI would be a possible therapeutic approach for cancer chemotherapy.

## Additional files

Additional file 1: **Figure S1.** Additive effects of GPR119 agonists on cell proliferation inhibition by gefitinib in breast cancer cell lines. (A, B) Additive effect of GSK1292263 (GSK) on cell proliferation inhibition by gefitinib in MCF-7 (A) and MDA-MB-231 cells. MCF-7 and MDA-MB-231 cells were treated with 10 μM gefitinib and 1–10 μM GSK (left), or GSK alone (right). (C) Additive effects of MBX-2982 (MBX) on cell proliferation inhibition by gefitinib in SK-BR-3 and MDA-MB-468 cells. (D) Combined effect of gefitinib with oleoylethanolamine (OEA) on cell proliferation of MCF-7 cells. The cells were incubated with 10 μM gefitinib in the presence or absence of 10 mM OEA. Relative proliferation rate was calculated by Incucyte® ZOOM basic analyzer. Data represent the mean ± S.D. (*n* = 6)(*** *p* < 0.005, significant difference between the indicated two groups). (PDF 119 kb)

Additional file 2: **Figure S2.** Identification of cellular metabolites with significant differences (*p* < 0.05) in MBX-2982-treated MCF-7 cells. MCF-7 cells were treated with 10 μM MBX-2982 for 24 h, and the relative cellular contents of metabolites were determined by LC/Ms./Ms. Data represent the mean ± S.D. (*n* = 3). (PDF 118 kb)

Additional file 3: **Figure S3.** Roles of GPR81 and NDRG3 in autophagy inhibition by MBX-2982. (A) GPR81 mRNA expression in GPR81 shRNA-infected cells. MCF-7 cells were infected with shGPR81 or shNonTarget lentivirus particle, and GPR81 mRNA expression was determined by real-time qPCR. Data represent the mean ± S.D. (n = 3)(*** *p* < 0.005, significant difference versus shNonTarget infected control). (B) Effect of GPR81 shRNA on autophagy inhibition by MBX-2982. LC3B I/II were determined by immunoblotting in shRNA control or shGPR81-infected MCF-7 cells. Both the cells were treated with 10 μM gefitinib in the presence or absence of 10 μM MBX-2982 for 24 h. Data represent the mean ± S.D. (n = 3)(* *p* < 0.05, significant difference between the indicated two groups). (C) Protein expression of NDRG3 (intracellular lactate receptor). MCF-7 cells were treated with 10 μM gefitinib in the presence or absence of 10 μM MBX-2982 for 24 h, and proten level of NDRG3 was monitored by immunoblotting. (D) Spheroid formation assay. 10^3^ MCF-7 cells were incubated with 10 μM gefitinib and/or 10 μM MBX-2982 for 96 h on ULA plate. Left, Representative spheroid images. Right, Spheroid volume. Data represent the mean ± S.D. (n = 3)(** *p* < 0.01, significant difference between the indicated two groups). (PDF 119 kb)

Additional file 4: **Figure S4.** Mitochondrial distribution of MBX-2982 and complex I inhibition. (A) GPR119-independent inhibition of OCR by 10 μM MBX-2982. OCR and ECAR were measured by XFp analyzer in both non-target shRNA- or GPR119 shRNA-infected MCF-7 cells. Both the cell types were treated with MBX-2982 (1–10 μM). (B) Mitochondrial complex I inhibition by MBX-2982. Complex I inhibition by MBX-2982 (10^− 10^, 10^− 8^ and 10^− 6^ M) was tested by mitostress test kit. Rotenone (100 nM) was used as a positive control of complex I inhibition. (C) Relative amounts of MBX-2982 were determined by LC/Ms./Ms. MCF-7 cells were incubated with MBX-2982 (10 μM) for 6 h and then homogenized. Cellular component fraction was isolated by sucrose gradient method. (PDF 107 kb)

## Abbreviations

3-MA: 3-methyladenine
4EBP1: Eukaryotic translation initiation factor 4E-binding protein 1
ACC: Acetyl-CoA carboxylase
AMPK: AMP-activated protein kinase
ATG3: Autophagy related 3
ATG5: Autophagy related 5
ATG7: Autophagy related 7
CRE: cAMP responsive element
ECAR: Extracellular acidification rate
EGFR: Epidermal growth factor receptor
GPCRs: G-protein coupled receptors
LC3B: Microtubule associated protein 1 light chain 3 beta
LDH: Lactate dehydrogenase
MCT1: Monocarboxylate transporter 1
MCT2: Monocarboxylate transporter 2
MCT4: Monocarboxylate transporter 4
mTOR: mechanistic target of rapamycin kinase
NDRG3: N-myc downstream regulated gene member 3
NMR: Nuclear magenetic resonance
NSCLC: Non-small cell lung cancer
OCR: Oxygen consumption rate
OXPHOS: Oxidative phosphorylation
p70S6K: Ribosomal protein S6 kinase B1
PARP: Poly(ADP-ribose) polymerases
PBS: Phosphate-buffered saline
PI: Propidium iodide
PI3K: Phosphatidylinositol-4,5-bisphosphate 3-kinase
PKA: Protein kinase A
TEM: Transmission electron microscopy
TKIs: Tyrosine kinase inhibitors
TNBC: Triple negative breast cancer
ULA: Ultra-low attachment
ULK1: Unc-51 like autophagy activating kinase 1
