# Correction: GPR119 agonist enhances gefitinib responsiveness through lactate-mediatedinhibition of autophagy

**DOI:** 10.1186/s13046-022-02388-0

**Published:** 2022-05-17

**Authors:** Ji Hye Im, Keon Wook Kang, Sun Young Kim, Yoon Gyoon Kim, Yong Jin An, Sunghyouk Park, Byung Hwa Jung, Song-Yi Choi, Jin-Sun Lee, Keon Wook Kang

**Affiliations:** 1 College of Pharmacy and Research Institute of Pharmaceutical Sciences, Seoul National University, Seoul, Republic of Korea; 2 Department of Nuclear Medicine, College of Medicine, Seoul National University, Seoul, Republic of Korea; 3 College of Pharmacy, Dankook University, Cheonan-si, Republic of Korea; 4 Natural Product Research Institute, College of Pharmacy, Seoul National University, Seoul, Republic of Korea; 5 Molecular Recognition Research Center, Korea Institute of Science and Technology, Seoul, Republic of Korea; 6 College of Medicine, Chungnam National University, Daejeon, Republic of Korea

**Correction: J Exp Clin Cancer Res 37, 295 (2018)**

**https://doi.org/10.1186/s13046-018-0949-2**

Following publication of the original article [1], the authors identified an error in the author name of the 7^th^ author, Byung Hwa Jung.

The incorrect author name is: Byung Hwa Jeong

The correct author name is: Byung Hwa Jung

The author group has been updated above.
