# Supplementary figures and images for: GPR119 agonist enhances gefitinib responsiveness through lactate-mediated inhibition of autophagy
Source: J Exp Clin Cancer Res. 2018 Nov 29;37:295. doi: 10.1186/s13046-018-0949-2 (PMC6267899; doi:10.1186/s13046-018-0949-2)

Figure S1

A

MCF-7

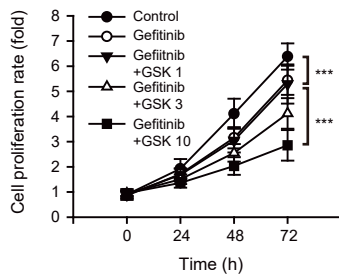

B

MBA-MB-231

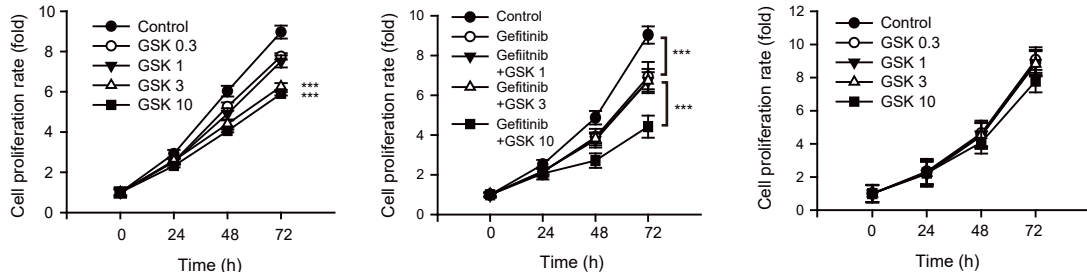

C

SK-BR-3

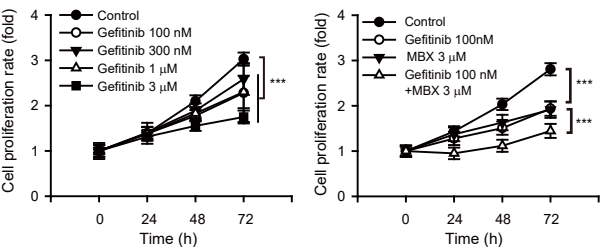

MDA-MB-468

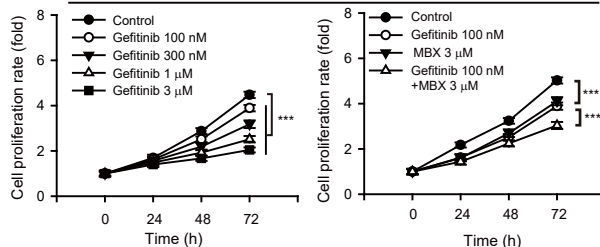

D

MCF-7

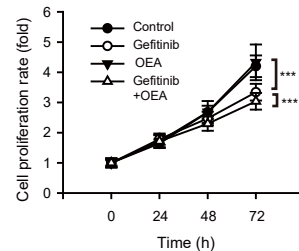

Supplement: Supplementary file 1 — Figure S1. Additive effects of GPR119 agonists on cell proliferation inhibition by gefitinib in breast cancer cell lines. (A, B) Additive effect of GSK1292263 (GSK) on cell proliferation inhibition by gefitinib in MCF-7 (A) and MDA-MB-231 cells. MCF-7 and MDA-MB-231 cells were treated with 10 μM gefitinib and 1–10 μM GSK (left), or GSK alone (right). (C) Additive effects of MBX-2982 (MBX) on cell proliferation inhibition by gefitinib in SK-BR-3 and MDA-MB-468 cells. (D) Combined effect of gefitinib with oleoylethanolamine (OEA) on cell proliferation of MCF-7 cells. The cells were incubated with 10 μM gefitinib in the presence or absence of 10 mM OEA. Relative proliferation rate was calculated by Incucyte® ZOOM basic analyzer. Data represent the mean ± S.D. (n = 6)(*** p < 0.005, significant difference between the indicated two groups). (PDF 119 kb) [file 13046_2018_949_MOESM1_ESM.pdf]

Figure S2

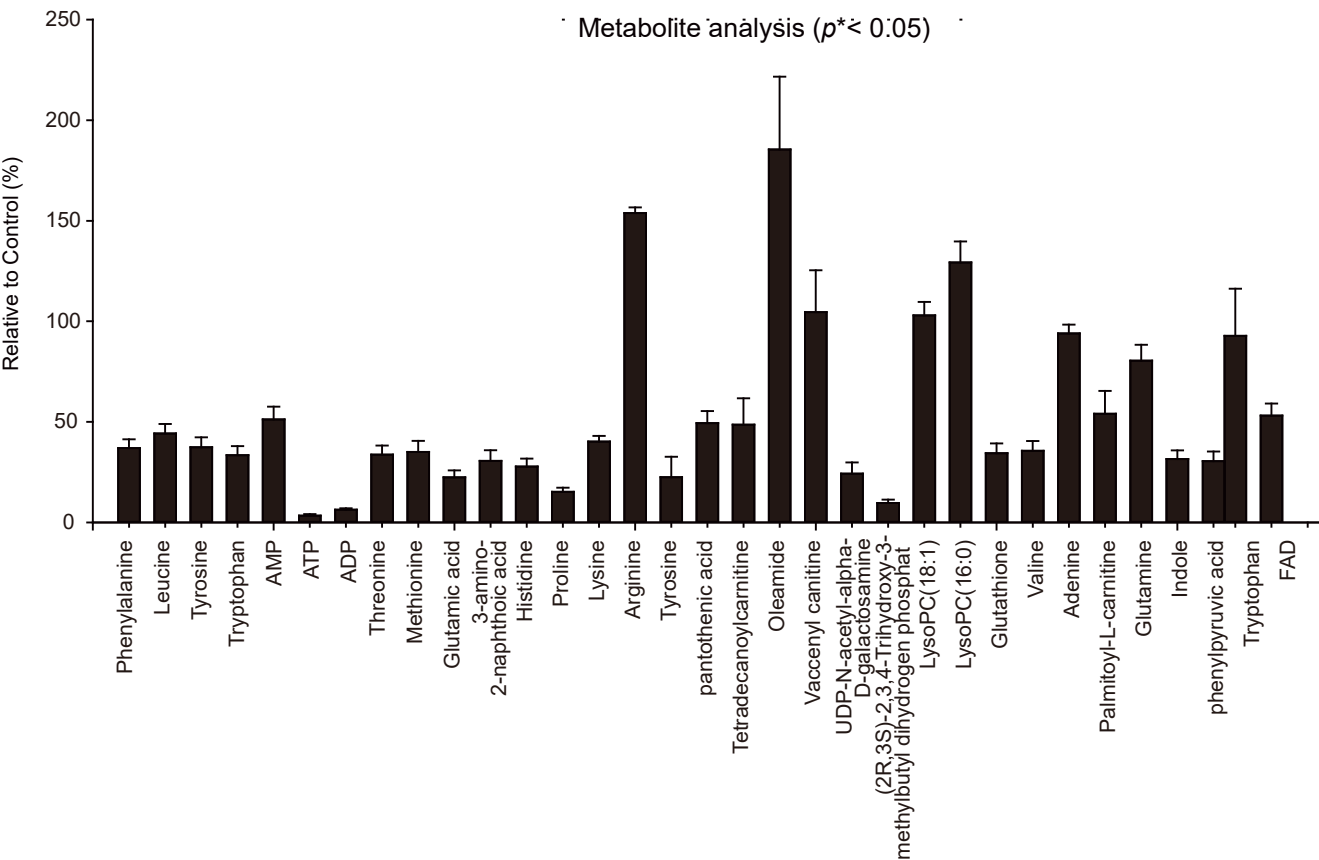

Supplement: Supplementary file 2 — Figure S2. Identification of cellular metabolites with significant differences (p < 0.05) in MBX-2982-treated MCF-7 cells. MCF-7 cells were treated with 10 μM MBX-2982 for 24 h, and the relative cellular contents of metabolites were determined by LC/Ms./Ms. Data represent the mean ± S.D. (n = 3). (PDF 118 kb) [file 13046_2018_949_MOESM2_ESM.pdf]

Figure S3

A

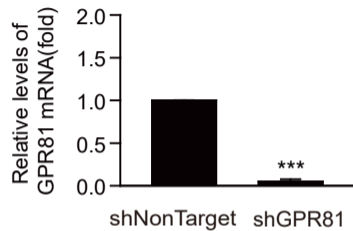

B

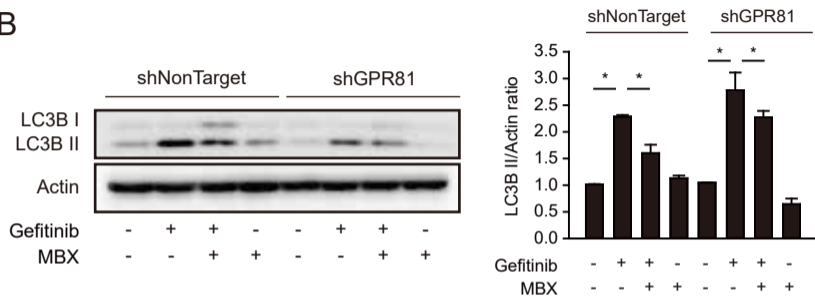

C

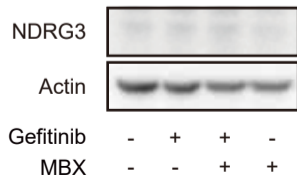

D

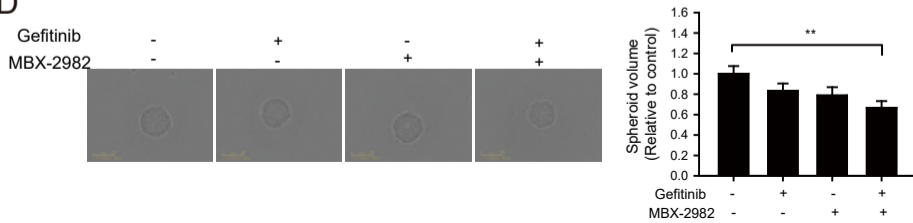

Supplement: Supplementary file 3 — Figure S3. Roles of GPR81 and NDRG3 in autophagy inhibition by MBX-2982. (A) GPR81 mRNA expression in GPR81 shRNA-infected cells. MCF-7 cells were infected with shGPR81 or shNonTarget lentivirus particle, and GPR81 mRNA expression was determined by real-time qPCR. Data represent the mean ± S.D. (n = 3)(*** p < 0.005, significant difference versus shNonTarget infected control). (B) Effect of GPR81 shRNA on autophagy inhibition by MBX-2982. LC3B I/II were determined by immunoblotting in shRNA control or shGPR81-infected MCF-7 cells. Both the cells were treated with 10 μM gefitinib in the presence or absence of 10 μM MBX-2982 for 24 h. Data represent the mean ± S.D. (n = 3)(* p < 0.05, significant difference between the indicated two groups). (C) Protein expression of NDRG3 (intracellular lactate receptor). MCF-7 cells were treated with 10 μM gefitinib in the presence or absence of 10 μM MBX-2982 for 24 h, and proten level of NDRG3 was monitored by immunoblotting. (D) Spheroid formation assay. 103 MCF-7 cells were incubated with 10 μM gefitinib and/or 10 μM MBX-2982 for 96 h on ULA plate. Left, Representative spheroid images. Right, Spheroid volume. Data represent the mean ± S.D. (n = 3)(** p < 0.01, significant difference between the indicated two groups). (PDF 119 kb) [file 13046_2018_949_MOESM3_ESM.pdf]

Figure S4

A

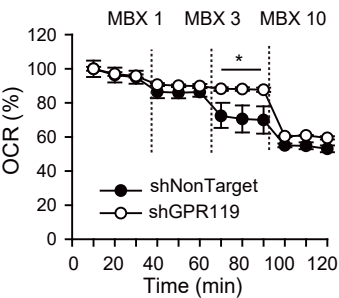

B

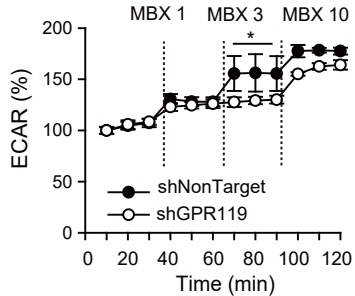

C

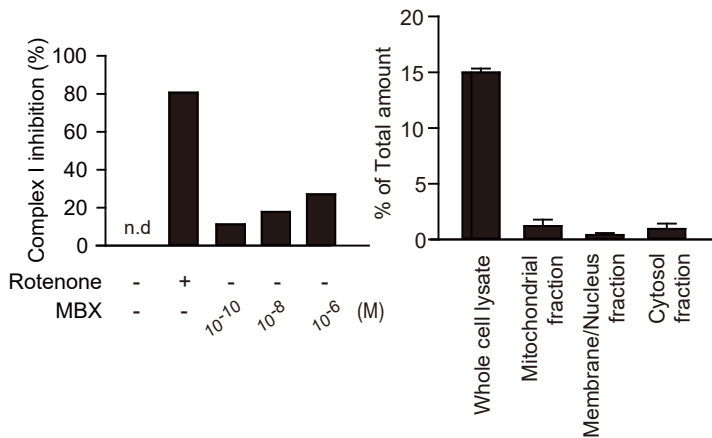

Supplement: Supplementary file 4 — Figure S4. Mitochondrial distribution of MBX-2982 and complex I inhibition. (A) GPR119-independent inhibition of OCR by 10 μM MBX-2982. OCR and ECAR were measured by XFp analyzer in both non-target shRNA- or GPR119 shRNA-infected MCF-7 cells. Both the cell types were treated with MBX-2982 (1–10 μM). (B) Mitochondrial complex I inhibition by MBX-2982. Complex I inhibition by MBX-2982 (10− 10, 10− 8 and 10− 6 M) was tested by mitostress test kit. Rotenone (100 nM) was used as a positive control of complex I inhibition. (C) Relative amounts of MBX-2982 were determined by LC/Ms./Ms. MCF-7 cells were incubated with MBX-2982 (10 μM) for 6 h and then homogenized. Cellular component fraction was isolated by sucrose gradient method. (PDF 107 kb) [file 13046_2018_949_MOESM4_ESM.pdf]
